# Cell instructive Liquid Crystalline Networks for myotube formation

**DOI:** 10.1016/j.isci.2021.103077

**Published:** 2021-09-02

**Authors:** Daniele Martella, Michele Mannelli, Roberta Squecco, Rachele Garella, Eglantina Idrizaj, Diego Antonioli, Michele Laus, Diederik S. Wiersma, Tania Gamberi, Paolo Paoli, Camilla Parmeggiani, Tania Fiaschi

**Affiliations:** 1 Istituto Nazionale di Ricerca Metrologica INRiM, 10135 Turin, Italy; 2 European Laboratory for Non-Linear Spectroscopy (LENS), University of Florence, 50019 Sesto Fiorentino, Italy; 3 Department of Biomedical, Experimental, and Clinical Sciences "Mario Serio", University of Florence, 50143 Florence, Italy; 4 Department of Experimental and Clinical Medicine, University of Florence, 50134 Florence, Italy; 5 Dipartimento di Scienze e Innovazione Tecnologica, Università del Piemonte Orientale “A. Avogadro”, 15121 Alessandria, Italy; 6 Department of Physics and Astronomy, University of Florence, 50019 Sesto Fiorentino, Italy; 7 Department of Chemistry “Ugo Schiff”, University of Florence, 50019 Sesto Fiorentino, Italy

**Keywords:** Cell, Tissue engineering, Biomaterials

## Abstract

Development of biological tissues *in vitro* is not a trivial task and requires the correct maturation of the selected cell line. To this aim, many attempts were done mainly by mimicking the biological environment using micro/nanopatterned or stimulated scaffolds. However, the obtainment of functional tissues *in vitro* is still far from being achieved. In contrast with the standard methods, we here present an easy approach for the maturation of myotubes toward the reproduction of muscular tissue. By using liquid crystalline networks with different stiffness and molecular alignment, we demonstrate how the material itself can give favorable interactions with myoblasts helping a correct differentiation. Electrophysiological studies demonstrate that myotubes obtained on these polymers have more adult-like morphology and better functional features with respect to those cultured on standard supports. The study opens to a platform for the differentiation of other cell lines in a simple and scalable way.

## Highlights

• Liquid crystalline network are able to induce myogenesis of C2C12 myoblasts
• Myogenesis is obtained without surface modifications or external stimulation
• Mature myotube physiological features are obtained on liquid crystalline networks

## Graphical abstract

**Figure:**
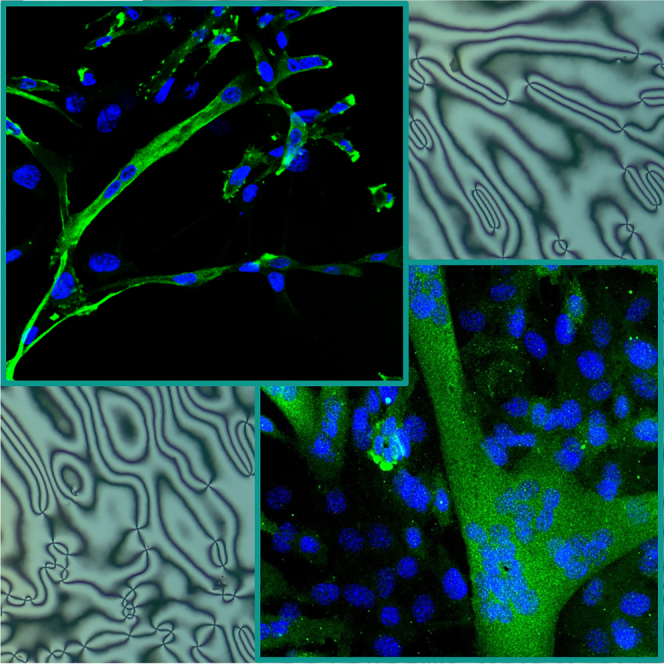

## Introduction

The availability of materials used as scaffolds to support cell proliferation and differentiation is one of the main requirements in tissue engineering. These materials should enable the replication of biological morphogenetic pathways, necessary for tissue formation *in vitro,* and adapt their physicochemical properties to mimic the natural environment of a selected cell line. The study of the complex relationship between scaffolds and cells is generally referred to as materiobiology and many examples of materials and composites mimicking the extracellular matrix (ECM) environment have been reported to date (Li et al., 2017).

For what skeletal muscle engineering is concerned, myotubes deriving from the fusion and differentiation of myoblasts must be engineered in linear arrays to replace the native muscle structure (Koning et al., 2009). Such linearized structures can be replicated *in vitro* by scaffolds bearing linear protrusions. Different micropatterned polymers are currently known to guide the aligned growth of myoblasts (Altomare et al., 2010; Gingras et al., 2009), and the technique is nowadays generalized for many kinds of cells, from fibroblast to cardiomyocytes (Pioner et al., 2019).

Moving toward reproduction of the ECM morphology, interesting examples include scaffolds consisting in nano fibers arrays prepared by electrospinning with a wide range of materials from biodegradable polycaprolactone or collagen (San Choi et al., 2008) to composites as gelatin-carbon nanotube mixtures (Ostrovidov et al., 2014). In some cases, the cell alignment induced by the anisotropic electrospun array has been demonstrated also to be responsible for the improvement of myoblast differentiation (Ricotti et al., 2012).

The introduction of an external stimulation during the culture can be also used to induce the differentiation process. For example, electric fields or static magnetic fields can be applied to enhance the total myo-nuclear density and myoblast differentiation (Quigley et al., 2012; Coletti et al., 2007). Alternatively, unidirectional cyclic stretching can be used to mimic the native dynamic tissue microenvironment and align the cells (Ergene et al., 2019). However, all these approaches introduce further factors to be controlled and complicate the protocols for the cell culture, thus limiting the scalability of such biological experiments up to real medical laboratories.

To overcome these limitations, a new option is to design materials that induce a correct cellular differentiation without having features similar to natural ECM. Development of these simple biomaterials aims to use the same standard biological protocol (e.g. without any application of stimuli) and to avoid complex scaffold fabrication toward a wide applicability of the procedure. In this case, the scaffold has to act as cell instructive material able to induce specific cellular response without any other trick or operation.

We recently reported that Liquid Crystalline Networks (LCNs) can be used as scaffolds for different cell lines, demonstrating superior properties with respect to commercial materials (Martella and Parmeggiani, 2018). In particular, human-induced pluripotent stem cell-derived cardiomyocytes (hiPSC-CMs) develop more adult-like morphology and functional cells than on standard Petri dishes, where hiPSC-CMs grow with a circular shape and totally aberrant function (Martella et al., 2017b).

One of the key properties of LCNs is their anisotropic molecular structure that confers extraordinary features such as shape-changing behavior under stimuli (as light irradiation or heating) (Ohm et al., 2010). Their potential applications range from the preparation of artificial muscles (Ferrantini et al., 2019), to the fabrication or soft robots on different length scales (Martella et al., 2019c; Wani et al., 2017; Cheng et al., 2010) and even optical devices (Nocentini et al., 2018; Guo et al., 2020). LCNs can be prepared starting from commercial acrylate-based mesogens incorporating the liquid crystalline units inside a polymeric backbone (White and Broer, 2015). The resulting anisotropic molecular structure is reflected in many macroscopic properties such as optical (Martella et al., 2019b) and mechanical anisotropic properties (Ware et al., 2016).

Starting from simple flat LCN materials, we demonstrated that C2C12 myoblasts grow with a unidirectional alignment that reflects the molecular arrangement and the overall orientational degree can be increased by modulating the polymer stiffness (Martella et al., 2019d). Later, other research groups demonstrated that LCN self-assembled structures can be exploited for the alignment of different cell lines due a spontaneous surface roughness/pattern formation (Turiv et al., 2020; Babakhanova et al., 2020; Jiang et al., 2020). However, the capability of LCNs in inducing muscle differentiation has not yet been deeply investigated and this study results mandatory since the process of cell alignment and their fusion/differentiation are regulated by different biological processes.

In this paper, we explored LCNs as cell instructive biomaterials for myogenesis. Using C2C12 murine myoblasts, we demonstrated that both the material stiffness and the mesogen molecular alignment strongly influence differentiation of C2C12 myoblasts. Our results show that LCNs characterized by medium stiffness and prepared starting from mesogens with homogeneous planar or isotropic alignment improve myotube formation. Interestingly, the myotubes formed on LCNs show better functional features in comparison to myotubes formed on commercial substrates (Petri dish or glass) as revealed by some of the key electrophysiological properties. These findings open the possibility to use such LCNs for the growth and differentiation of satellite cells that are muscle stem cells which are very difficult to obtain with adult features culturing them on standard supports for long periods.

## Results and discussion

### Scaffold preparation and characterization

**Figure 1:**
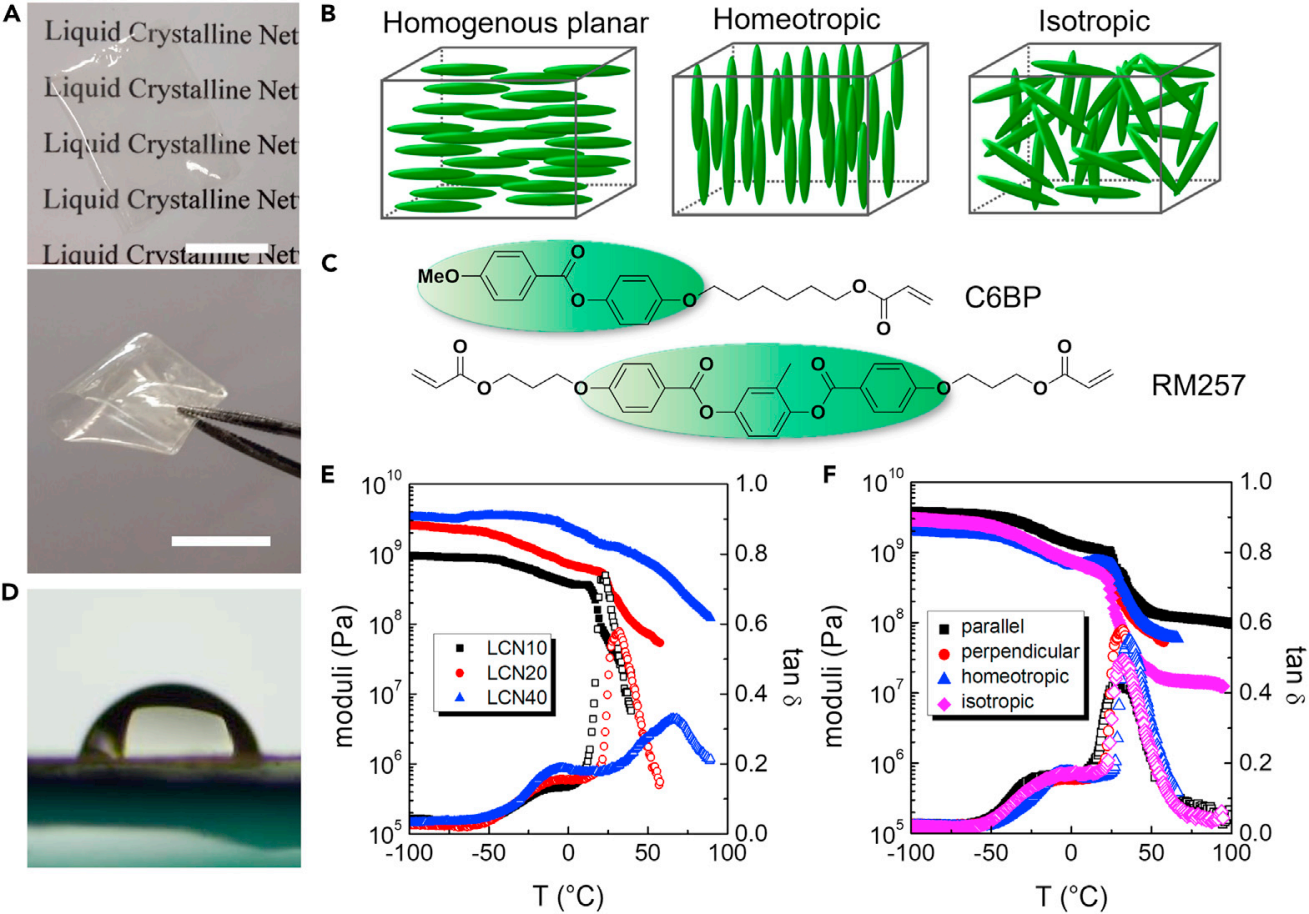
Material tested as cell scaffolds (A) optical images of LCN films (scale bars: 1 cm); (B) scheme of the different LC alignments tested; (C) molecular structure of the monomers; (D) water contact angle measurement on LCN20; (E) DMA on films with different crosslinker content and homogeneous planar alignment: trend of E′ and tan δ (measured in perpendicular direction with respect to the LC alignment) as a function of temperature; (F) DMA on LCN20 films with different alignments: trend of E′ and tan δ for as a function of temperature in homogeneous planar (in both parallel and perpendicular direction with respect to the director), homeotropic and isotropic orientations (E′ closed symbols and tan δ open symbols).

Polymeric scaffolds have been fabricated as transparent and flexible films of centimeter length scale (Figure 1A). The materials presented a nematic order (with directors oriented in different ways) or an isotropic conformation (Figure 1B) and they were prepared by photopolymerization of a mixture of two acrylate based mesogens (C6BP and RM257) whose structures are reported in Figure 1C. All monomeric mixtures showed a nematic liquid crystalline (LC) phase on cooling that was aligned to form a monodomain texture before the polymerization (Martella et al., 2017b). In particular, the monomers were infiltrated in an LC cell composed by two glasses coated with different sacrificial layers able to induce homogeneous planar or homeotropic alignment. After annealing in the nematic phase, a monodomain alignment was obtained and the material was irradiated by UV light causing the polymer formation (with the retention of the molecular alignment set before this step). To be noted that the final properties of the LCNs are strictly dependent on both monomer composition and polymerization conditions, e.g. the temperature set during irradiation can modify the macroscopic properties of the films (Hebner et al., 2021)

The materials tested in this study are called LCNx, where x (=10, 20 or 40) represent the % mol/mol of the cross-linker (RM257) present in the formulation. Increasing the amount of RM257 leads to a higher cross-linking degree thus influencing the overall material performances. We focused both on nematic (homogeneous planar or homeotropic) and isotropic films (a sketch of the molecular alignments is depicted in Figure 1B). In homogeneous planar samples, molecules are aligned in the rubbing direction being parallel to the film surface, while in homeotropic one mesogens tend to arrange perpendicular to the surface. In the last case, the material is prepared starting from monomers in their isotropic arrangements and during the irradiation, no change in the material texture was observed thus excluding the formation of polydomain structures. All the formulations have hydrophilic characteristics with water contact angle around 70° (Figure 1D) and slightly varying in dependence of the cross-linker content (Martella et al., 2019d). Also the surface roughness has been previously characterized and is barely dependent from the cross-linker concentration (Martella et al., 2019d).

The samples bearing a homogeneous planar alignment were characterized also in terms of order parameter (S) by polarized absorption spectroscopy (Figure S1). S slightly decreases by increasing the cross-linker amount with values of 0.62, 0.61, and 0.56 for LCN10, LCN20, and LCN40 respectively.

On the other hand, the ratio in between the monomers is very important also to modulate the elastic modulus and the damping capabilities of the final material (Martella et al., 2017a, 2019a). To highlight this fundamental aspect (mechanical modulus is one of the main characteristics to modulate to design a cell scaffold), a dynamic mechanical analysis (DMA) has been performed on films prepared with the three formulations (LCN10, LCN20 and LCN40) presenting a homogeneous planar alignment. In Figure 1E, mechanical analysis is reported for measurement done in the perpendicular direction with respect to the LC director. In the glassy state, dynamic storage modulus E′ increases as a function of the cross-linker amount (Martella et al., 2017a), while it decreases as a function of temperature, and a drop of one order of magnitude is observed around the glass transition of various samples. In the rubbery state, the increases of the cross-linking degree lead to higher E′ while the glass transition temperature also increases and becomes broader (Hikmet and Broer, 1991). A second relaxation process (β transition) partially overlapped to the glass transition is recorded for all samples, and it can be attributed to relaxation of the mesogenic units (Martella et al., 2017a, 2019a; Hikmet and Broer, 1991). Measurements performed in the parallel direction follow a similar trend (Figure S2A) and show higher E′ values with respect to perpendicular direction whereas lower tan δ values are observed thus indicating a less lossy behavior (Figure S2B). Also the molecular alignment influences the mechanical properties (Hikmet and Broer, 1991), and this effect has been evaluated for the samples LCN20 having different orientations. In addition to homogeneous planar alignment (both in parallel and perpendicular direction), homeotropic or isotropic arrangements were analyzed in Figure 1F. Trends of E′ and tan δ as a function of temperature for homeotropic and isotropic arrangements are quite similar to those observed for homogeneous ones. In the glassy state, dynamic storage modulus for isotropic alignment is intermediate with respect to parallel and perpendicular orientations, whereas E′ for homeotropic alignment is overlapped on the perpendicular one. On the other hand, samples prepared from mesogens in the isotropic phase present the lower storage modulus in the rubbery state which can be the result of the different polymerization condition applied (irradiation at 90°C with respect to 45°C used for all the aligned LC films) rather than an effect of the mesogen alignment.

### Differentiation of myoblasts on liquid crystalline scaffolds

**Figure 2:**
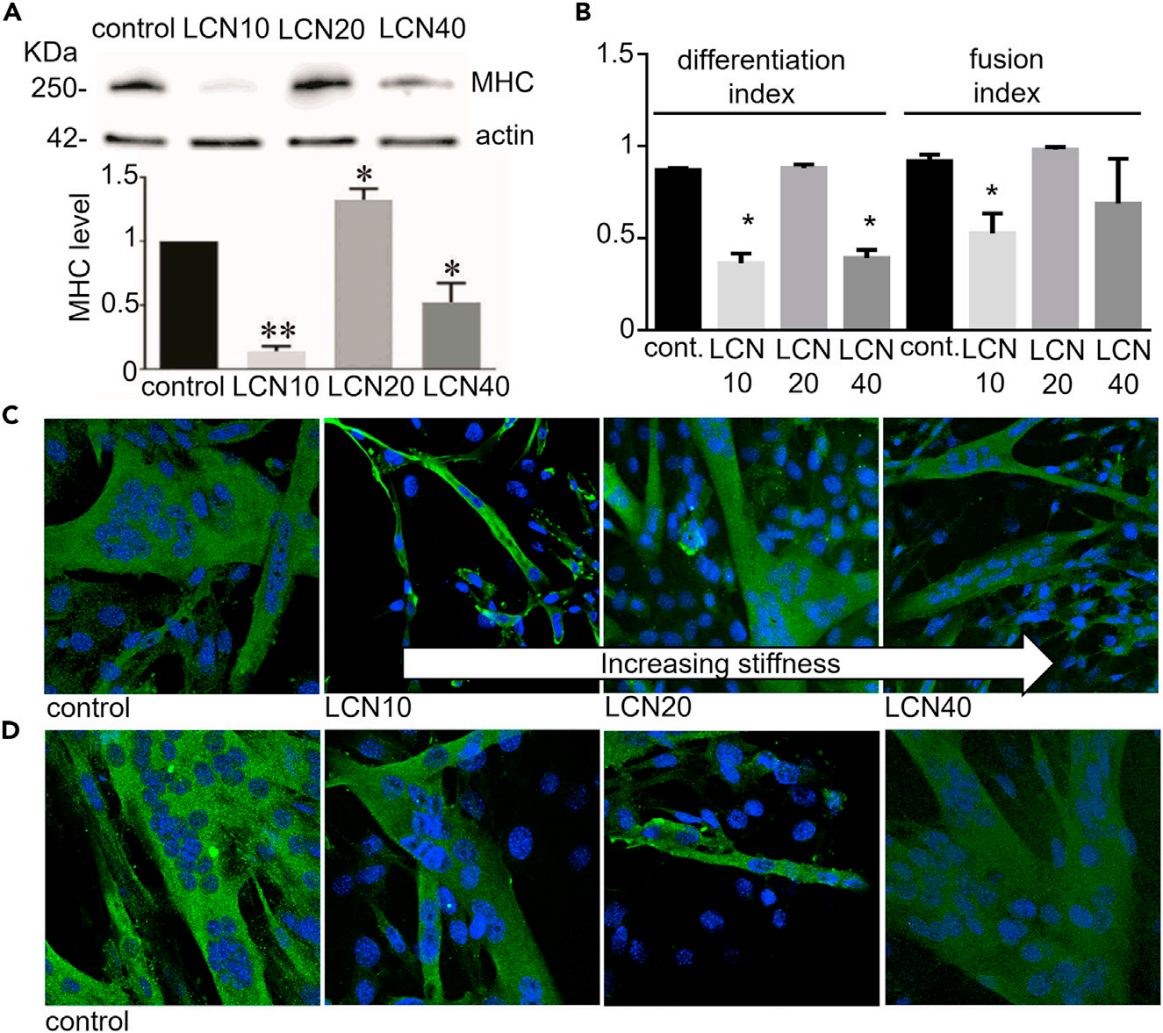
Differentiation of myoblasts on LCN (A) Immunoblot analysis of Myosin Heavy Chain (MHC). Myoblasts were differentiated on LCNs with different stiffness and homogeneous planar alignment for four days. Control myotubes are formed on Petri dishes (or glass coverslips for the confocal analysis). Bar graph shows MHC level obtained by ImageJ, using actin immunoblot for normalization, considering control as 1. (B) Differentiation and fusion indexes for myotubes formed as described in (A). (C) Representative images of myotubes obtained by confocal analysis. Myotubes are formed as described in (A). (D) Representative images of myotubes obtained by confocal analysis. Myotubes are formed on LCN20 with different molecular orientations. Green staining shows MHC, while blue color evidences the nuclei. The same results were obtained in three independent experiments. ∗p < 0.05 *vs* C, ∗∗p < 0.001 *vs* (C)

The biological tests were focused on the ability of C2C12 myoblasts to differentiate into myotubes on the different LCNs. First, C2C12 myoblasts have been plated on materials having different cross-linker percentages and characterized by a homogeneous planar orientation. C2C12 myoblasts have been also seeded in Petri dishes used as control. The results show that the LCN20 constitutes the best substrate for C2C12 myoblast differentiation with myotube formation comparable to that obtained on a Petri dish. This effect can be observed by optical images (Figure S3A), measurement of myotube width (Figure S3B) and the expression level of MHC (muscle-type myosin heavy chain), a marker of muscle differentiation, evaluated by immunoblot analysis (Figure 2A). Conversely, LCN40 induces a worst differentiation compared to LCN20, while LCN10, having less stiffness, drastically blocks myogenesis (Figure 2A). Similar results were obtained by confocal microscope analysis as reported in Figure 2C, showing myotubes obtained on the different LCNs. The images, in which MHC is highlighted in green, whereas the nuclei are stained in blue, clearly showing that the LCN20 allows for the formation of the best myotubes, as demonstrated by higher differentiation and fusion indexes (Figure 2B). We also analyzed the number of the total nuclei on the different LCNs after four days of differentiation, as shown in Figure S3C. Nuclei count was an estimation of cell viability on the LCNs throughout the experiment. After plating myoblasts at the same density on LCNs, we observed that LCN10 contains the lower number of nuclei in comparison to LCN20 and LCN40. These data suggest that LCN10 induces myoblast death during differentiation, and LCN40 inhibits the correct muscle differentiation without affecting cell viability, whereas LCN20 permits the survival of the majority of the myoblasts which then differentiate into myotubes. To further study the features of myotubes, we analyzed myotube length formed on LCNs and Petri dishes. The result, shown in Figure S3D, demonstrated that myotubes formed on LCN20 resulted longer in comparison to those formed on LCN10, LCN40 and control substrate. Collectively, our findings demonstrate that LCN20 is an excellent substrate for myogenesis, since it promotes the survival of the majority of myoblasts, thus inducing the formation of myotubes having the highest MHC level (Figure 2A) with the greater width (Figure S3B) and length (Figure S3D) with respect to the other LCNs and to commercial substrate.

To better elucidate how the molecular arrangement affects the differentiation of myoblasts, the same analysis has been performed using LCN20 presenting different LC alignments. Confocal analysis shows adequately formed myotubes on homogeneous and isotropic LCN20 (Figure 2D) and this observation is confirmed by differentiation index reported in Figure S4A. Myotubes formed on homogeneous and isotropic LCN20 show a differentiation index similar to control, while in homeotropic LCN20 the value is statistically lower with respect to control myotubes. Conversely, the fusion index is not influenced by the LCN alignment (Figure S4A). The analysis of the nuclei amount on different LCNs demonstrated that cell viability is not affected by homogeneous or isotropic alignment, whereas homeotropic one decreases the number of nuclei on LCN (Figure S4B). Also the myotube length (Figure S4C) is reduced on homeotropic scaffolds.

These results demonstrated that both homogeneous and isotropic alignment induces the correct myotube formation with respect to homeotropic alignment. Interestingly, such behavior is not dependent only by the mechanical properties of the substrate (see Figure 1F), since similar stiffness gives different biological responses, thus suggesting for other molecular parameters to play a pivotal role. We can speculate that different alignments would result in a different exposure of functional groups affecting the cell-polymer interaction and then inducing a different degree of myogenesis. This hypothesis should explain the different cell behavior on homogeneous planar and homeotropic LCN 20 samples, where stiffness is very similar, and deserve to be better explored in the near future.

### Electrophysiological characterization of myoblasts and myotubes

**Figure 3:**
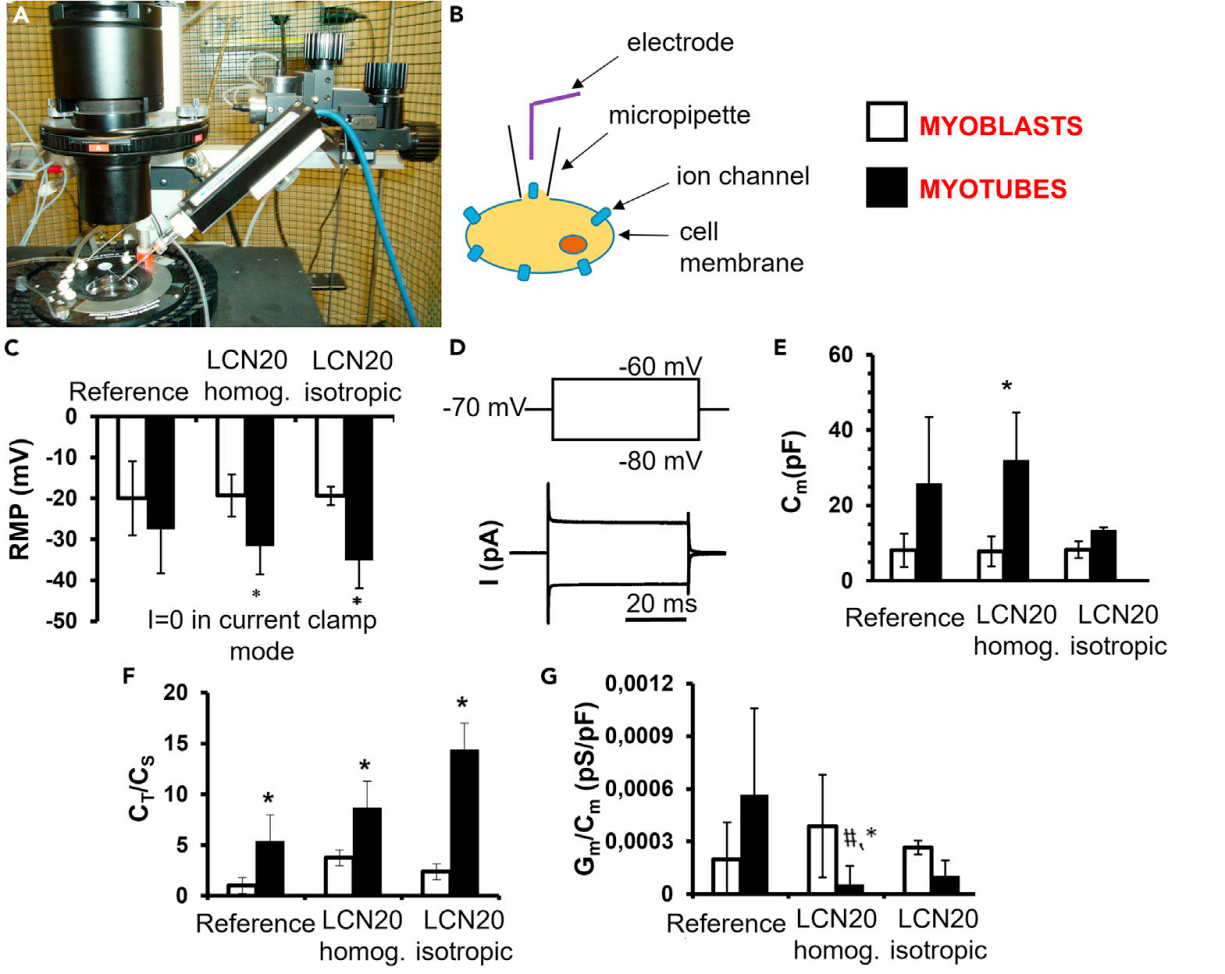
Electrophysiological records (A) image of experimental chamber and holder with patch pipette; (B) Scheme for the whole cell patch clamp; (C) resting membrane potential (RMP, in mV) evaluated in current clamp mode with a stimulus I = 0 nA in myoblasts (open bars) and myotubes (filled bars), cultured in the three conditions tested; (D) pulse protocol of stimulation (top) and typical tracings of passive membrane currents (bottom) obtained in response to its application in voltage clamp condition; (E) cell capacitance (C_m,_ in pF) as an index of cell surface; (F) ratio C_T_/C_s_ as an index of T tubular development; (G) specific membrane permeability, G_m_/C_m_ (pS/pF). Data are mean ± SD. ∗p < 0.05 vs the corresponding myoblast population, #p < 0.05 vs myotubes on reference substrate (one way ANOVA followed by Bonferroni's *post hoc* test). The analysis was made on at least three independent experiments.

To further demonstrate the capability of LCNs to work as cell instructive scaffolds for myogenic maturation, we investigated some typical electrophysiological features suitable to demonstrate the acquisition of a more mature phenotype in myotubes. In particular, we evaluated the materials selected in the previous experiments (LCN20 with homogeneous planar and isotropic alignment), by using the equipment shown in Figures 3A and 3B. First, we considered the features usually related to the differentiation state, such as the resting membrane potential (RMP) and the membrane passive properties. A summary of the different passive membrane properties is reported in Table S1. Since the hyperpolarization of the skeletal muscle cell membrane potential is suggested to be a trigger for its differentiation (Tanaka et al., 2017), we expected that the myotubes tended to be more hyperpolarized than myoblasts in any condition tested. However, RMP measured in myotubes formed on reference substrate did not significantly differ from that measured in the related myoblasts (Figure 3C), whereas the observed hyperpolarization was statistically significant for myotubes formed on LCNs if compared with the related myoblasts. This observation suggests that LCN20 is a better substrate for myotubes to gain a major control of their resting potential and to lessen the impact of non-selective ion fluxes (leakage currents and so on), thus leading to an optimal asset of excitability.

We then estimated the cell membrane capacitance (C_m_) that provides an index of cell surface width, as well as information about the membrane chemistry (Muratore et al., 2012). The analysis of C_m_ on all our cells indicates an expected increase in myotubes compared with the related myoblasts in any condition tested but statistically significant differences have been found only in myotubes formed on homogeneous LCN20 (Figures 3D and 3E). In addition, aiming to assess if the observed increase of C_m_ was due also to an effective enlargement of the T-tubular system (occurring mature skeletal muscle fibers) (Carozzi et al., 2000), we calculated the C_T_/C_s_ ratio, where C_T_ represents the T-tubular capacitance and C_s_ the surface (sarcolemnic) capacitance (Squecco et al., 2009). The ratio resulted significantly increased in myotubes with respect to myoblasts and this rise became more evident for myotubes formed on LCN20 (Figure 3F) suggesting the involvement of the C_T_ value enhancement. The resting membrane permeability (G_m_ value) did not show statistically significant differences in any condition (data not shown). However, myotubes formed on homogeneous LCN20 showed the lowest G_m_/C_m_ value (Figure 3G) suggesting that the specific membrane conductance significantly decreases during myogenesis mainly due to the great increase of cell surface area. In addition, since the membrane permeability measured in resting condition may be also due to the opening state of stretch activated channels (SAC), the progressive decline of G_m_/C_m_ observed is in line with previous studies reporting that SAC expression is higher in proliferating myoblasts than in differentiated myotubes. In fact, the permeability of these non voltage-dependent channels is critical in the early differentiation process of myogenic cells (Formigli et al., 2009) and decreases under differentiation (Formigli et al., 2007).

**Figure 4:**
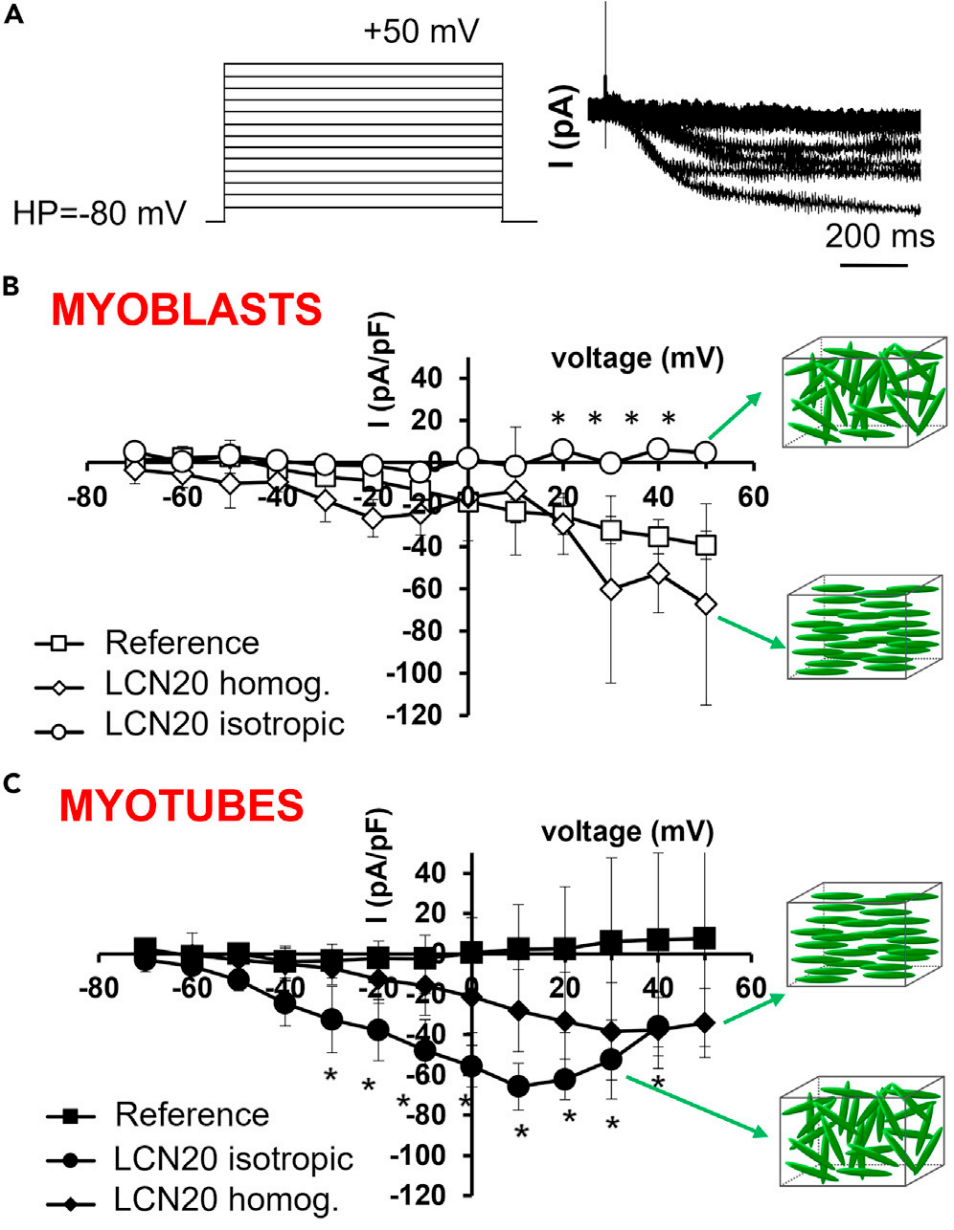
Measure of transmembrane ion current (A) Pulse protocol of stimulation (left) and typical tracings of inward ion currents obtained in response to its application in voltage clamp condition (right). I-V plot of Calcium currents evoked in myoblasts (B) and in myotubes (C) in the three different culture conditions Data are mean ±SD. ∗Indicates p<0.05 vs the reference substrate (two way ANOVA followed by Bonferroni’s *post hoc* test). The analysis was made on at least three independent experiments.

At the end, we analyzed the transmembrane ion currents flowing through voltage-dependent Ca^2+^ channels (I_Ca_), known to play a key role in excitation-contraction (EC) coupling in adult skeletal muscle fibers (Shimahara and Bournaud, 1991). When stimulated with the voltage pulse protocol shown in Figure 4A C2C12 myoblasts cultured on the reference substrate elicited a small-amplitude inward current (Figure 4B), as expected for undifferentiated cells, starting from a voltage threshold of about −30/-20 mV. In particular, the I-V plot showed that the current amplitude increased almost linearly as the applied voltage step increased, suggesting a scarcely voltage dependent ion entry. In contrast, myoblasts plated on homogeneous planar LCN20 (Figure 4B) showed an inward current with a similar amplitude but with a lower voltage threshold of activation at about −50/-40 mV, possibly indicating the T-type Ca^2+^ current occurrence. The I-V plot showed in this case two maximal current values: one at −20 mV and another at about +30 mV, suggesting the presence of two different voltage-dependent phenomena, the second of which could presumably be the L-type Ca^2+^ current. Finally, myoblasts grown on isotropic LCN20 (Figure 4B) did not show any appreciable inward current.

The analysis of the I-V plot related to the differentiated myotubes showed different results (Figure 4C). The inward current was very small in amplitude for myotubes maintained on the reference substrate. Conversely, myotubes formed on homogeneous planar LCN20 (Figure 4C) show increased inward current, ranging from a voltage threshold of about −40 mV to the maximal amplitude at +30 mV and showing inactivation for voltage steps higher of +30 mV, suggesting the involvement of L-type Ca^2+^ channels. However, the inward current amplitude shows larger values in myotubes cultured on isotropic LCN20 with a voltage threshold of about −60/-50 mV, a maximal current evoked by the +10 mV pulse and two different slopes for negative step pulses, suggesting the involvement of two different phenomena in this overall inward current. Again, we propose the occurrence of voltage-dependent Ca^2+^ currents in myotubes cultured on isotropic LCN20 that resemble the T and L-type ones. Since the L-type Ca^2+^ current is a key event for EC coupling in mature skeletal muscle, and this appears to be prevalent in myotubes cultured on isotropic LCN20, we propose this substrate as the best candidate to gain the acquisition of functional voltage dependent Ca^2+^ channels useful for an adult-like skeletal muscle EC coupling.

### Limitations of the study

We demonstrated that Liquid Crystalline Networks are good cell instructive materials for myotube formation. The electrophysiological tests allow us to enlighten the functional aspect of myoblast-to-myotube differentiation and clearly indicate LCN20 as the best candidate to reach the goal compared with a reference substrate (the commonly used Petri dishes or glass cover-slips). Furthermore, the electrophysiological analysis resulted in the evidence that isotropic scaffolds are more suitable to obtain functional myotubes, since these supports result in higher L-type Ca^2+^ currents of the myotubes.

However, further studies are needed to clarify the role of mesogen alignment and, in particular, if this aspect can lead to different functional groups exposure that influences the biological response. Also the influence of other material parameters needs to be further investigated by our future research. To be noted that an improved myogenesis is obtained on materials presenting a higher stiffness than those of biological muscles (with LCN elastic moduli in the MPa range), thus suggesting a range of optimal stiffness suitable to improve differentiation. The use of the materials toward a closer natural tissue stiffness deserves to be investigated. This first analysis aims to speed up on the obtainment of functional muscular tissues *in vitro*, but it is limited to C2C12 murine cells. In the near future, our approach would be applied toward a plethora of cell cultures and tissues. Furthermore, the presented technology looks to be even suitable for culturing satellite muscle stem cells, very difficult to obtain mature on standard supports, improving their growth and leading to functional tissues.

## STAR★Methods

### Key resources table

**Table:** 

REAGENT or RESOURCE	SOURCE	IDENTIFIER
**Antibodies**		

Anti-MHC primary antibodies	Santa Cruz Biotechnology	sc-376157
Anti-actin primary antibodies	Santa Cruz Biotechnology	sc-47778
Goat anti-mouse Ig-G HRP secondary antibodies	Santa Cruz Biotechnology	sc-2005
Anti-mouse secondary antibodies Alexa Fluor 488	Invitrogen	A32766

**Chemicals, peptides, and recombinant proteins**		

Monomer R257	Synthon Chemicals GmbH & Co.	CAS: 1174063-87-7
Monomer C6BP	Synthon Chemicals GmbH & Co.	CAS: 130953-14-9
Irgacure 369	Merck KGaA	CAS: 119313-12-1
Poly(vinyl alcohol)	Merck KGaA	CAS: 9002-89-5
Polyimide Varnish SUNEVER	Nissan Chemical Corporation	Grade 1211 Type 0626
Disperse Red 1 (DR1)	Merck KGaA	CAS: 2872-52-8
4′,6-diamidino-2-phenylindole (DAPI)	Sigma-Aldrich	D9542
PVDF membrane	Biorad	10026933

**Critical commercial assays**		

Bradford assay	Biorad	5000006

**Experimental models: Cell lines**		

C2C12 murine myoblasts	Laboratory of Paolo Porporato, University of Turin, Italy	N/A

**Software and algorithms**		

ChemBioDraw Ultra 12.0	PerkinElmer Inc.	https://perkinelmerinformatics.com/products/research/chemdraw/
Excel 2016	Microsoft corporation, Washington, USA	https://www.microsoft.com
Pclamp 6.0	Axon Instruments Foster City, CA, USA	https://www.moleculardevices.com/products/axon-patch-clamp-system/acquisition-and-analysis-software/
Clampfit 9.0	Axon Instruments Foster City, CA, USA	https://www.moleculardevices.com/products/axon-patch-clamp-system/
Image J	National Institutes of Health, USA	https://imagej.nih.gov/
Graph Pad	GraphPad Software Inc., CA, USA	https://www.graphpad.com/

**Other**		

A/D-D/A interfaces (Digidata 1200)	Axon Instruments, Foster City, CA	N/A
Pump 33	Harvard Apparatus LTD	Pump 33 DDS (Dual Drive System) Syringe Pump - Harvard https://www.harvardapparatus.com
Puller	Narishige, Tokyo, Japan	http://products.narishige-group.com
Axopatch 200B amplifier	Axon Instruments Foster City, CA	https://www.moleculardevices.com/products/axon-patch-clamp-system/amplifiers/axon-instruments-patch-clamp-amplifiers#gref
Borosilicate glass tubing - (GC150-7.5) Standard wall, without filament, 1.5 x 75 mm (OD x L)	Harvard apparatus LTD	Cat#30-0056
Universal holder, 1.0-1.7 diameter	Molecular Devices	Cat#1-HL-U

### Resource availability

#### Lead contact

Further information and requests for resources and reagents should be directed to and will be fulfilled by the lead contact, Daniele Martella (d.martella@inrim.it).

#### Materials availability

This study did not generate new unique reagents.

### Experimental model and subject details

#### Cell line

The animal cell line used in this study belong to murine C2C12 myoblasts (organism: mus musculus) and it has been provided by Dr. P. Porporato (University of Turin, Italy).

C2C12 cells have culture in DMEM culture medium supplemented with 10% fetal bovine serum at 37°C and 5% CO_2_. Differentiation has been carried on for four days in DMEM containing 2% horse serum at 37°C and 5% CO_2_.

### Methods details

#### Scaffold fabrication and characterization

Liquid crystalline monomers C6BP and RM257 were purchased by Synthon Chemical, while the photoinitiator Irgacure 396 was purchased from Sigma-Aldrich and used as received.

##### Film preparation

All monomeric mixtures contained the three compounds with C6BP in 79-59% mol/mol, RM257 in 20-40% mol/mol and the photoinitiator Irgacure 369 in 1% mol/mol. The mixtures were melted in isotropic phase (above 65°C) and then infiltrated by capillarity in appropriate polymerization cells. After cooling down to 45°C (to reach the desired monodomain alignment), the sample was irradiated for 10 min with a UV LED lamp (Thorlabs M385L2-C4, 385 nm, I = 1.8 mW cm^−2^) and then, for further 10 minutes at 65°C. Isotropic samples were polymerized directly at 90°C after the infiltration step. The cells were manually opened and the films removed by a blade and used for the cell culture without further purification. Before the cell culture tests, LCNs have been sterilized by three washes in 70% ethanol for 15 minutes followed by four washes in PBS. LCNs have been dried for one hour under a sterile hood and then transferred to a Petri dish or a glass.

The polymerization cells were composed of two glasses separated by some 20 μm sized spheres as spacers. Depending on the desired LC alignment, different sacrificial layers were used as coating for the glasses. In particular, to reach the homogenous planar alignment, the cell was prepared with glasses coated with a PVA solution (1% w/w in water) and rubbed unidirectionally with a velvet cloth. On the other hand, for homeotropic alignment, the glasses were spin coated with the polyimide PI1211 (Nissan Chemicals Industries).

##### Order parameter characterization

The order parameter (S) was estimated by polarized absorption spectroscopy for LCNs with homogeneous planar alignment and containing 0.5% in weight of Disperse Red 1. Spectra were recorded on a Varian Cary 400 instrument and the dichroic ratio (R) was evaluated at the dye absorption maximum wavelength (500 nm) according to equation: R = (A_∥_)/(A_⊥_), where A_∥_ and A_⊥_ represent the parallel and perpendicular absorbance with respect to liquid crystalline director (Figure S1). The order parameter (S) was defined by the equation: S = (R-1)/(R+2).

##### Mechanical test

Dynamic-Mechanical Thermal Measurements (DMTA) were performed in tensile mode using a DMTA V analyzer (Rheometric Scientific). Specimens with size of 20 × 5 × 0.02 mm^3^ were employed. The measurements were carried out using a strain amplitude of 0.1% (linear viscoelastic range), a strain frequency of 1 Hz and a scanning rate of 4°C/min.

#### C2C12 culture and differentiation

C2C12 murine myoblasts were provided by Dr. P. Porporato, University of Turin, Italy. Unless differently specified, all reagents were obtained from Sigma-Aldrich, Inc.; SDS-PAGE materials were from Bio-Rad Laboratories; anti-Myosin Heavy Chain (MHC) and anti-actin primary antibodies were from Santa Cruz; Alexa 488 fluorescent secondary antibodies were from Pierce. ECL detection reagents were from Bio-Rad Laboratories.

##### Cell culture

Murine C2C12 myoblasts were cultured in growing medium composed of Dulbecco's modified Eagle's medium (DMEM) supplemented with 10% fetal bovine serum in 5% CO_2_ humidified atmosphere. For differentiation, sub-confluent C2C12 were shifted from growing to differentiating medium composed of DMEM containing 2% Horse Serum (HOS). *Confocal analysis.* C2C12 myoblasts were grown until sub-confluence on glass coverslips (used as control) or LCN and then differentiated for four days. Myotubes were washed with PBS and fixed in 3% paraformaldehyde for 20 min at 4°C. Fixed cells were permeabilized with three washes with TBST (50 mM Tris–HCl, pH 7.4, 150 mM NaCl, 0.1% Triton X-100), and then blocked with 5.5% HOS in TBST for 1 h at room temperature. Cells were then incubated with specific primary antibodies, diluted 1:100 in TBS (50 mM Tris–HCl, pH 7.4, 150 mM NaCl), overnight at 4°C. Cells were then washed once with TBST and once with TBST with 0.1% BSA and then incubated with Alexa 488 secondary antibodies (diluted 1:100) for 1 h at room temperature in TBST with 3% BSA. For the staining of nuclei 4′,6-diamidino-2-phenylindole (DAPI) 10 μM final, was added to TBST and the cells were treated for five minutes at room temperature. After extensive washes in TBST, cells were mounted with glycerol plastine. In all experiments emitted fluorescence was analyzed using a confocal fluorescence microscope (Leica TCS SP8).

##### Nuclei staining

Myoblasts were plated at the same density on each substrate and the day after, the differentiating medium was added. After four days of differentiation the nuclei were stained with 4′,6-diamidino-2-phenylindole (DAPI), 10 μM final, and visualized under a confocal fluorescence microscope (Leica TCS SP8).

##### Differentiation and fusion indexes and analysis of myotube width

The differentiation index has been calculated as the percentage of Myosin Heavy Chain positive cells above the total nuclei. The fusion index is the average number of nuclei in Myosin Heavy Chain positive cells (containing at least three nuclei) above the total nuclei. Myotube width was measured by ImageJ. Ten randomly chosen fields for each sample were analyzed and the average value was reported in the bar graph.

##### Immunoblot analysis

Cells were lysed for 20 min on ice in 500 μl of complete radio-immunoprecipitation assay (RIPA) buffer. Lysates were clarified by centrifugation, and total protein contents were obtained using Bradford assay (Bio-Rad Laboratories). 20 μg of total proteins for each sample were separated by SDS-PAGE and transferred onto PVDF membranes. PVDF membranes were incubated in 2% milk, probed with primary antibodies, and incubated with secondary antibodies conjugated with horseradish peroxidase. Quantification of bands was achieved using ImageJ software.

#### Electrophysiological characterization

The electrophysiological features of C2C12 cells were investigated by the whole cell patch-clamp technique in current- and voltage-clamp conditions (Meacci et al., 2010; Squecco et al., 2016).

##### Set up and solutions

During the whole cell patch clamp recordings, cells plated on the different substrates were continuously superfused at a rate of 1.8 mL min^−1^ with a physiological bath solution having the following composition (mM):150 NaCl, 5 KCl, 2.5 CaCl_2_, 1 MgCl_2_, 10 D-glucose and 10 HEPES. pH was titrated to 7.4 with NaOH. The patch pipettes were obtained using a vertical puller (Narishige, Tokyo, Japan) from borosilicate glass tubing (Harvard apparatus LTD) and were filled with an internal solution containing (mM): 130 KCl, 10 NaH_2_PO_4_, 0.2 CaCl_2_, 1 EGTA, 5 MgATP and 10 HEPES that was filtered through 0.22 μm pores. pH was set to 7.2 with TEA-OH. To record only I_Ca_ (Idrizaj et al., 2018), we used a high-TEA external solution (mM): 10 CaCl_2_, 145 tetraethylammonium bromide (TEABr), 10 HEPES, and a suitable filling pipette solution (mM): 150 CsBr, 5 MgCl_2_, 10 EGTA, and 10 HEPES (pH = 7.2). The patch pipettes resistance ranged between 2 and 7 MΩ. The patch pipette was connected to a micromanipulator and an Axopatch 200 B amplifier (Axon Instruments, Foster City, CA) (Squecco et al., 2016; Meacci et al., 2010).

##### Pulse protocols of stimulation and data analysis

Current- and voltage-clamp protocol generation and data acquisition were controlled by using an output and an input of the A/D-D/A interfaces (Digidata 1200; Axon Instruments) and Pclamp 6 software (Axon Instruments Foster City, CA). The resting membrane potential (RMP) was measured by switching to the current clamp mode (I = 0) of the 200 B amplifier. We estimated the junction potential of the electrode (approximately −10 mV) before making the patch and then we subtracted this value from the recorded membrane potential. The membrane passive properties were evaluated in voltage clamp mode applying a 10-mV negative and positive step pulse starting from a holding potential (HP) of −70 mV (see Figure 3D) (Di Franco et al., 2016; Squecco et al., 2020). The decay of the elicited passive current (Squecco et al., 2009) could be best-fitted by the sum of two exponential functions, representing the time course of the sarcolemmal and tubular membrane currents, I_S_ and I_T_, respectively (Collins et al., 1982). The time constants associated with the current decay are τ_s_ = R_s_C_s_ and τ_T_ = R_T_C_T_, where R_s_ and R_T_ represent the resistances in parallel with C_s_ and C_T_, respectively; C_s_ is the capacitance related to the sarcolemmal membrane and C_T_ is that related to the tubular membrane. The ratio C_T_/C_s_ calculated in different conditions can provide an estimation of the relative contribution of C_T_ or C_s_, since this ratio increases with the numerator C_T_ (indeed, the ratio increases also when C_s_ decreases, but during differentiation this event is not expected since myoblasts fusion and myotube maturation is normally accompanied by cell growth, membrane enlargement and thus C_s_ augmentation). The membrane resistance (R_m_) was calculated from the relation: R_m_ = (ΔV − I_m_R_a_)/I_m_, where ΔV represents the command voltage step size, I_m_ is the steady-state membrane current and R_a_ is the access resistance (Squecco et al., 2015; Sassoli et al., 2011). The resting membrane conductance G_m_=1/R_m_ was considered an index of membrane permeability. We also calculated the specific membrane conductance by the ratio G_m_/C_m_, where C_m_ is the cell linear capacitance. The latter parameter was calculated from C_m_ = ΔQ(R_m_ + R_a_)/R_m_ΔV (Pappone and Lee, 1996) and is the overall result of C_s_ + C_T_. Notably, C_m_ was used as an index of the cell surface, since the membrane-specific capacitance is constant at 1 μF cm^-2^. Ion current activation was evoked from a HP=−80 mV, by applying 1 s long step voltage pulses, from −80 to 50 mV, in 10-mV increments. We used the P4 procedure to remove capacitive and leak currents. The currents were low-pass filtered with a Bessel filter at 2 KHz. To compare properly the current amplitudes elicited in cells of different dimensions we normalized their values to C_m_. Thus, the ratio I/C_m_ is intended as current density. The electrophysiological analysis was made on at least three independent experiments.

### Quantification and statistical analysis

#### Statistical analysis for differentiation and fusion indexes

Data are presented as mean ± S.D. from at least three independent experiments. Statistical analysis of the data was performed by Student’s t test or by one-way ANOVA using Graph Pad Prism version 6.0. p values < 0.05 were considered statistically significant.

##### Statistical analysis for electrophysiology

Data are collected from a representative, randomly selected portion of the total cell populations and the results of the experiments are expressed as mean ± SD. Student's unpaired t-test was used to compare the average values of two data sets, assuming that values follow a normal distribution. The one-way ANOVA was used for multiple comparisons followed by Bonferroni’s *post ho*c analysis. Statistical significance was set to p < 0.05.

## Data Availability

• All data reported in this paper will be shared by the lead contact upon request. • This paper does not report original code. • Any additional information required to reanalyze the data reported in this paper is available from the lead contact upon request.
