# Supplementary material for: Cell instructive Liquid Crystalline Networks for myotube formation
Source: iScience. 2021 Sep 2;24(9):103077. doi: 10.1016/j.isci.2021.103077 (PMC8449234; doi:10.1016/j.isci.2021.103077)
Supplement: Document S1. Figures S1–S4 and Table S1 [file mmc1.pdf]

iScience, Volume *24*

Supplemental information

Cell instructive Liquid Crystalline

Networks for myotube formation

Daniele Martella, Michele Mannelli, Roberta Squecco, Rachele Garella, Eglantina Idrizaj, Diego Antonioli, Michele Laus, Diederik S. Wiersma, Tania Gamberi, Paolo Paoli, Camilla Parmeggiani, and Tania Fiaschi

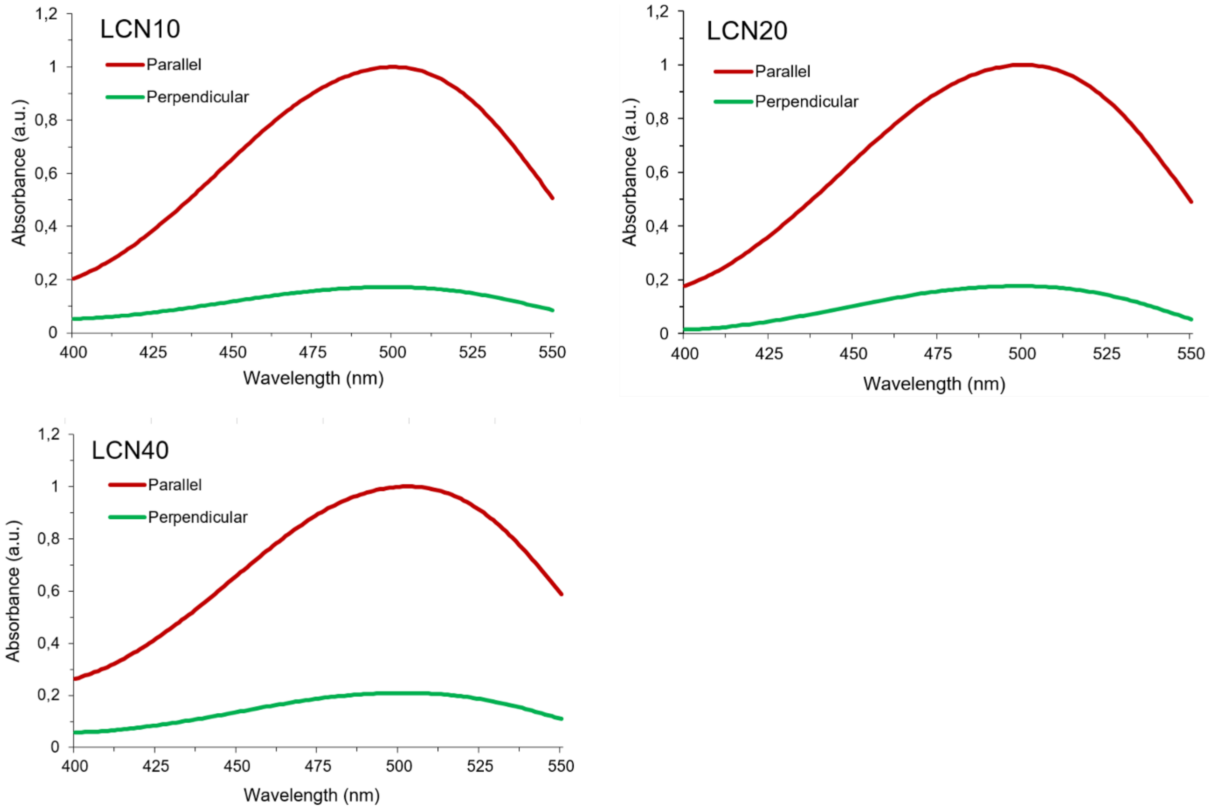

Figure S1. Polarized UV-vis absorption spectra of LCNs with planar homogeneous alignment. Spectra were recorded in parallel and perpendicular directions with respect to the nematic director. Related to STAR Methods.

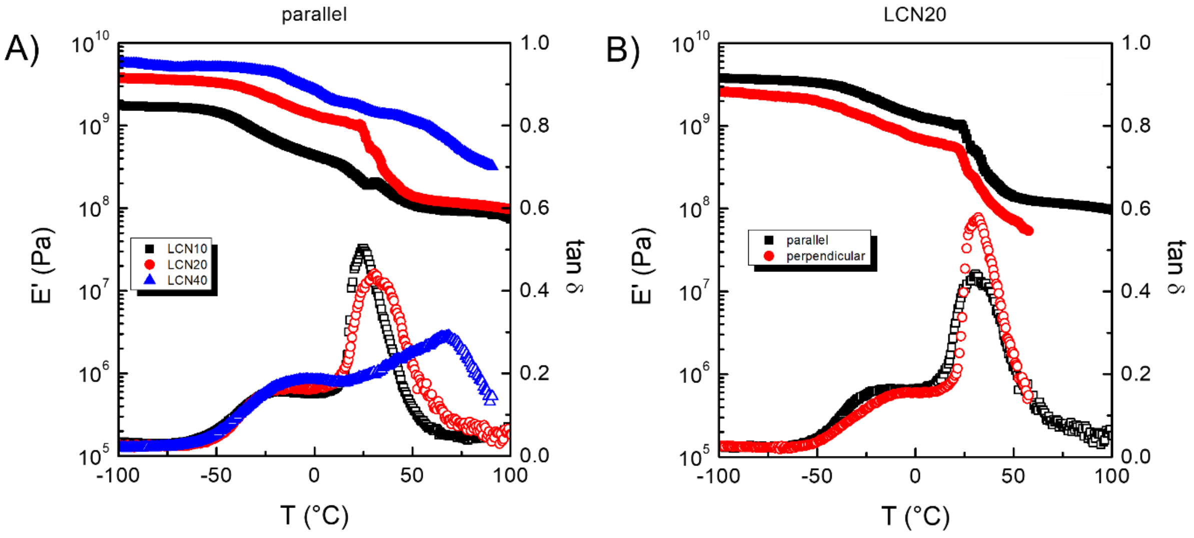

Figure S2. Additional DMA analysis. a) Trend of E' and tan δ in the homogeneous alignment (parallel direction with respect to the LC director) as a function of temperature for LCN10 (black), LCN20 (red) and LCN40 (blue) samples (E' fully symbols and tan δ open symbols). b) Trend of E' and tan δ in the homogeneous parallel (black) and perpendicular (red) alignment with respect to the liquid crystalline alignment as a function of temperature for LCN20 sample (E' full symbols and tan δ open symbols). Related to Figure 1.

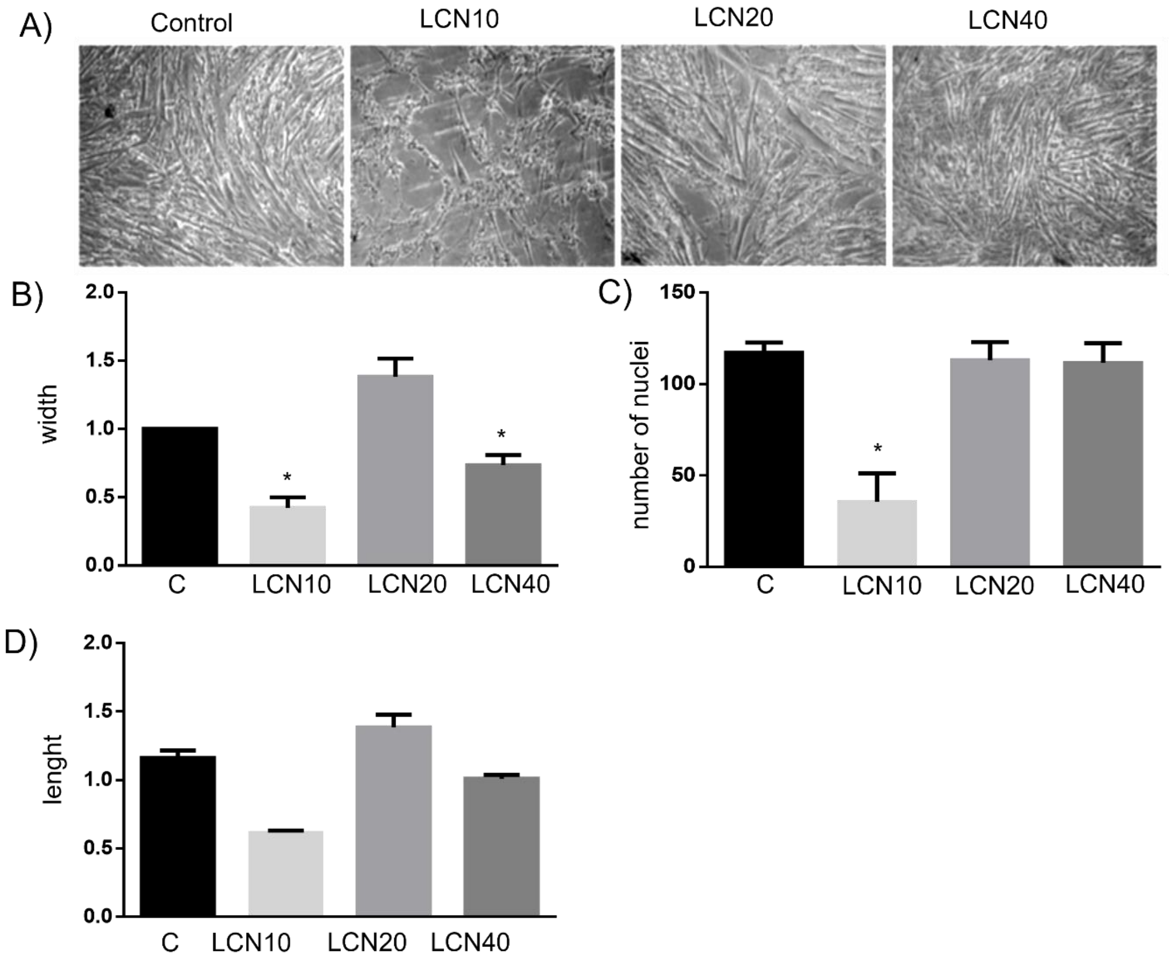

Figure S3. Analysis of myotubes formed on LCNs with homogeneous planar alignment and different stiffness. C2C12 myoblasts were plated on Petri dishes (Control, C) or LCNs until sub-confluence. Differentiation of myoblasts was carried on for four days. a) Representative images of myotubes, by optical microscope, obtained on Petri dishes (Control, C) and LCNs. b) Width of myotubes formed on different substrates. Ten randomly chosen fields are used for width measure by Image J and reported as mean in the bar graph. c) Number of nuclei counted after four days of differentiation. d) Myotube length. At least fifty randomly chosen myotubes have been used for the measure by Image J. The mean has been reported in the bar graph. The same results were obtained in three independent experiments. *$p<0.05$ *vs* C. Related to Figure 2.

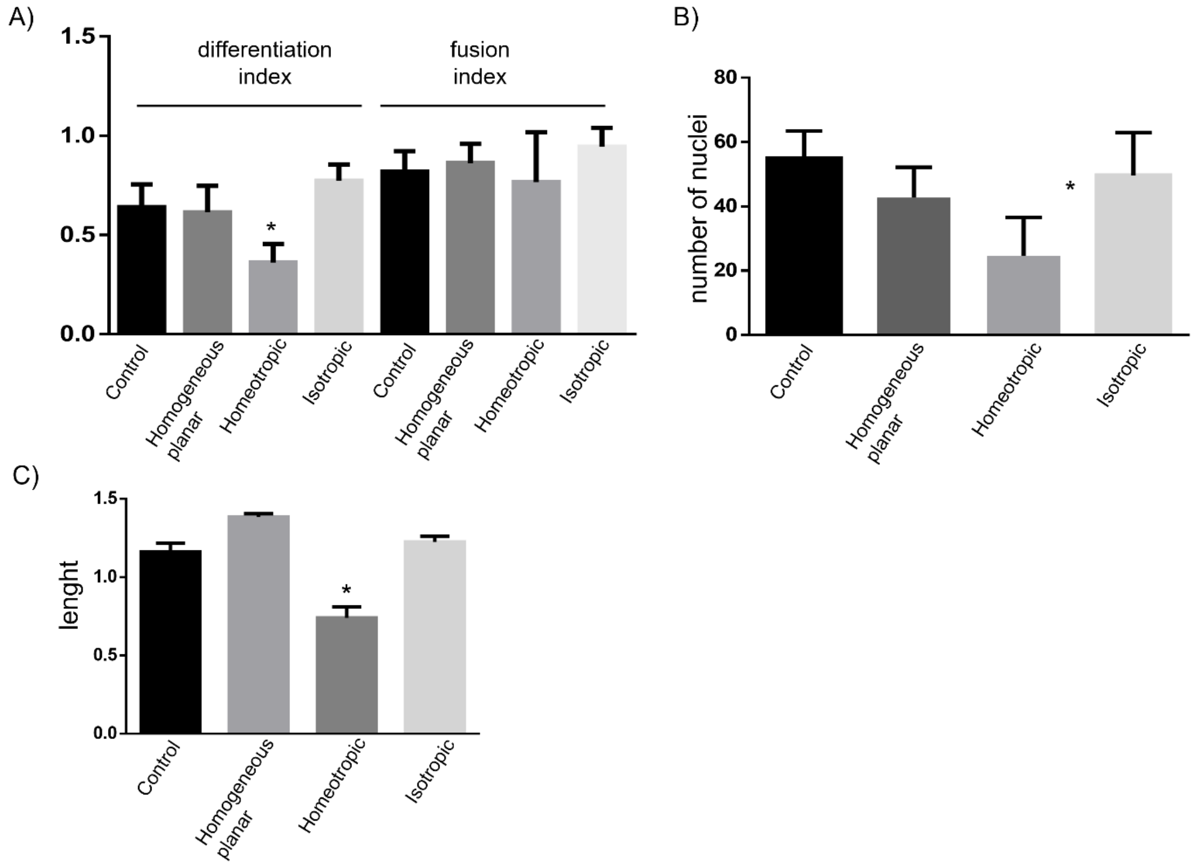

Figure S4. Analysis of myotubes formed on LCNs with same stiffness and different molecular alignment (namely, homogeneous planar, homeotropic and isotropic orientation). C2C12 myoblasts were plated on Petri dishes (Control) or LCNs until sub-confluence. Differentiation of myoblasts was carried on for four days. a) Differentiation and fusion indexes. b) Number of nuclei counted after four days of differentiation. c) Myotube length. At least fifty randomly chosen myotubes have been used for the measure by Image J. The mean has been reported in the bar graph. The same results were obtained in three independent experiments. *$p<0.05$ *vs* Control. Related to Figure 2.

Table S1. Passive membrane properties of myoblasts and myotubes in the different conditions. Related to Figure 3.

		Reference	LCN20 homogen.	LCN20 isotropic
RMP (mV)	Myobl	-20.1±6.4 (n=8)	-19.2±5.1 (n=6)	-18.1± 5.2 (n=10)
	Myot	-27.6±10.6 (n=10)	-31.6±6.9* (n=8)	-30.6± 6.9* (n=5)
C_m (pF)	Myobl	8.2±5.3 (n=11)	7.6±4.6 (n=10)	9.3±2.3 (n=10)
	Myot	25.8±17.6 (n=7)	39.9±12.7* (n=5)	16.0±3.7 (n=5)
C_T/C_S	Myobl	1.0±1.2 (n=2)	3.7±3.4 (n=5)	2.4±2.9 (n=3)
	Myot	5.3±5.0* (n=5)	8.6±8.6* (n=7)	14.3± 22.4* (n=3)
G_m/C_m (pS/pF)	Myobl	0.00027±0.0002 (n=7)	0.000308±0.0002 (n=10)	0.000265± 0.00006 (n=4)
	Myot	0.000563±0.0004 (n=12)	5.21E-05±0.0001*# (n=5)	0.000103± 0.00009 (n=5)

* p<0.05 *vs* related myoblasts cultured on the same substrate; # p<0.05 *vs* myotubes on reference substrate (one-way ANOVA with Bonferroni’s correction). n=number of cells included in the statistical analysis.
